# Micromorphological and histochemical attributes of flowers and floral reward in *Linaria vulgaris* (Plantaginaceae)

**DOI:** 10.1007/s00709-018-1269-2

**Published:** 2018-06-03

**Authors:** Jacek Jachuła, Agata Konarska, Bożena Denisow

**Affiliations:** Department of Botany, University of Life Sciences in Lublin, 15 Akademicka St., 20-950 Lublin, Poland

**Keywords:** Histochemical tests, Nectar and pollen rewards, Trichomes, Insect visitors

## Abstract

The self-incompatible flowers of *Linaria vulgaris* have developed a range of mechanisms for attraction of insect visitors/pollinators and deterrence of ineffective pollinators and herbivores. These adaptive traits include the flower size and symmetry, the presence of a spur as a “secondary nectar presenter,” olfactory (secondary metabolites) and sensual (scent, flower color, nectar guide—contrasting palate) signals, and floral rewards, i.e. pollen and nectar. Histochemical tests revealed that the floral glandular trichomes produced essential oils and flavonoids, and pollen grains contained flavonoids, terpenoids, and steroids, which play a role of olfactory attractants/repellents. The nectary gland is disc-shaped and located at the base of the ovary. Nectar is secreted through numerous modified stomata. Nectar secretion began in the bud stage and lasted to the end of anthesis. The amount of produced nectar depended on the flower age and ranged from 0.21 to 3.95 mg/flower (mean = 1.51 mg). The concentration of sugars in the nectar reached up to 57.0%. Both the nectar amount and sugar concentration demonstrated a significant year and population effect. Pollen production was variable between the years of the study. On average, a single flower of *L. vulgaris* produced 0.31 mg of pollen. The spectrum of insect visitors in the flowers of *L. vulgaris* differed significantly between populations. In the urban site, *Bombus terrestris* and *Apis mellifera* were the most common visitors, while a considerable number of visits of wasps and syrphid flies were noted in the rural site.

## Introduction

*Linaria vulgaris* (L.) Mill., a perennial herb, belongs to the family Scrophulariaceae, according to classic taxonomy systems (Cronquist 1981) or Plantaginaceae, according to the modern phylogeny system APG IV (Angiosperm Phylogeny Group). The species is native to temperate regions of Eurasia. However, it has also been successfully introduced to North America, Australia, New Zealand, and South Africa (CABI datasheet)*. L. vulgaris* is now considered as an invasive species in the USA and in Canada (Sing and Peterson 2011). The species occurs both in cultivated and uncultivated areas, but grows especially vigorously in disturbed habitats (Ward et al. 2009). The spread of *L. vulgaris* populations is due to efficient vegetative reproduction; the main stem is capable of forming up to 100 secondary shoots and surviving up to 4 years (Newman and Thomson 2005a). Sexual reproduction also occurs. *L. vulgaris* is considered to be an obligate outcrosser and evolved several adaptations towards attracting insects, e.g., well-noticeable flowers (Stout et al. 2000). However, the flowers of *L. vulgaris* are zygomorphic, deeply spurred, with closed access to the corolla throat (Fernández-Mazuecos et al. 2013). Such flowers are traditionally viewed as highly specialized (Stebbins 1970). Specialized flowers are however also visited by insect that do not fit “pollination syndrome” and pollinator composition may differ considerably between populations (even located closely to one another) (Nepi et al. 2003; de Merxem et al. 2009). Specific flower visitors are especially sensitive to changes in pollinating fauna induced by habitat types, i.e., functional group of insect to plant species can differ greatly between urban and suburban environments compared to semi-natural and agricultural ones (Geslin et al. 2013).

Flower specialization is a result of co-evolution between plants and specialized pollinators (Cacho et al. 2010). The plant-insect interactions are based on diverse signals perceived by insects, which have to learn to use different sensory channels to make the food search effective (Renner 2006). From the plant point of view, the floral shape and size, flower arrangements, color, and/or odor are important for advertisement of rewarding flowers ready for pollination (Fernández-Mazuecos et al. 2013; Sulborska et al. 2014; Balamurali et al. 2015).

Floral trichomes—hair-like epidermal structures—have been considered to play a role in plant-pollinator relations, e.g., signaling the pathway to the reward (Owen and Bradshaw 2011). In many *Linaria* species, corolla and calyx are covered with trichomes—glandular and/or non-glandular (Segarra and Mateu 2001; Saez and Crespo 2005). Petal trichomes may form clusters visible as a contrasting palate and signal the pathway to the reward (e. g., Owen and Bradshaw 2011). Glandular trichomes may produce different classes of secondary chemicals, which are stored or volatilized at the plant surface to attract pollinators and/or defend against non-effective insect visitors or even are important in plant resistance (Glas et al. 2012; Konarska 2017).

A crucial role in pollinator attraction is attributable to nectar and pollen, which are considered as main constituents in plant/animal interactions. In most angiosperms, nectar is an aqueous sugar-rich solution composed of three common sugars (sucrose, glucose, fructose) that acts as energetic reward for pollinators (Antoń and Denisow 2014; Denisow et al. 2016). The nectar parameters, nectar volume, sugar concentration, and relative composition of sugars vary widely across species (Chalcoff et al. 2017) and have an impact on food selection by insect visitors or pollinators (Baker and Baker 1983; Nicolson 2007; Rodríguez-Riaño et al. 2014). There is ample evidence that nectar production and the sugar concentration in nectar is an environmentally and physiologically related issue (e.g., Petanidou and Smets 1996). In *L. vulgaris*, numerous nectarostomata are involved in the exudation process. Nectar of *L. vulgaris* is composed mainly of sucrose, glucose, and fructose; however, trace amounts of rafinose have also been detected (Nepi et al. 2003). As nectar is gathered in the deep corolla spur, the nectar guides present in the corolla are supposed to increase the efficiency of the pollination process (Stout et al. 2000; Vargas et al. 2010).

Pollen is the main source of proteins, lipids, sterols, vitamins, and hormones and is a key component of balanced insect pollinator diet necessary to provide the required proportion of nutrients (Filipiak et al. 2017). There is weak evidence that some pollen traits (odor, protein content) may be attributed to whether the insect visitors/pollinators are inclined to collect pollen or nectar or both (Dobson and Bergström 2000; Pacini and Hesse 2005; Denisow et al. 2018).

The aim of this paper was to present floral features of *L. vulgaris* that can be attributable to the interaction with insect visitors. In particular, we (i) examined the flowering biology (phenology, anthesis length), (ii) identified the pattern of distribution of trichomes on the calyx and corolla, (iii) tried to recognize the main chemical classes of metabolites present in floral parts and pollen grains, and (iv) assessed the quantity of floral reward (nectar and pollen). In addition, we tried to evaluate whether the insect visitors are interested in *L. vulgaris* flowers; therefore, we made observations of the insect visitor activity and spectrum. We also examined whether there are differences in insect visitors composition in highly specialized flowers of *L. vulgaris* between two populations (rural and urban).

## Material and methods

### Study area

The field observations of *Linaria vulgaris* (L.) Mill. were carried out in 2013–2014 on Lublin Upland (51° 15′ 44 ′ N, 22° 30′ 48′ E, SE Poland). Two populations (separated by approx. 10 km) were selected for the experiment. The first population was grown in a rural area (in Jastków; R-population), and the second population originated from an urban area (in Lublin, U-population). Every year, we used plants from the same self-renewing population of the same experimental patches (approx. 4–6 m^2^ each). The plants of both populations were grown on loess soil at pH 6–7 in full-sun sites.

## Study methods

### Flowering and insect observations

The duration of flowering was recorded; the beginning of flowering was defined when 2–5% of flowers were in complete swelling and the end of flowering was identified when almost 90% of individuals finished blooming (=corolla wilted). The life span of an individual flower was defined as the period between lower lip folding (=the start of palate presentation) and corolla wilting. The intensity (=number) and spectrum of floral insect visitors were noted. Due to the long blooming period of the species, the observations of floral insect visitors were conducted in June, July, August, and September. In each period, the survey was performed for two to three consecutive days at 1-hour intervals between 5.00 and 18.00 (GMT + 2 h). Each census of observation was 5–10 min long. During the observations, the weather conditions were as follows: daily temperature above 10 °C, wind speed < 10 km h^−1^ with no precipitation. In the case of very strong wind or rain, the observations were ceased and completed on a subsequent day. All insect visitors were noted in each observation period. The observations of flowering and insect foraging were conducted in each population (rural—R and urban—U). Due to difficulties in taxonomic identification of insects in the field, only some insect visitors were identified to the species level. Insects were divided in several groups: *Apis mellifera*, *Bombus terrestris*, other *Bombus* spp. (including *B. lapidarius*, *B. hortorum*, *B. pascuorum*, and *B. sylvarum*), *Andrena* sp., *Vespula vulgaris*, syrphid flies, other dipterans, and Lepidoptera. The identification of *Bombus* spp. was based on Pawlikowski and Pawlikowski (2012).

### Microscopic examinations

In 2014, the floral microstructure was examined in 2–3 day of anthesis in light microscopy (LM), fluorescence light microscopy (FLM), and scanning electron microscopy (SEM). Samples for microscopic investigations were collected from flowers (*n* = 30) of different individuals (*n* = 10) from the rural and urban populations.

### Light microscopy

The height (at the highest point) and the external diameter of fresh nectaries (*n* = 10) as well as the length of different types of trichomes located on the calyx and corolla (*n* = 20) were measured.

### Histochemistry

Fresh hand-made sections of calyces and corollas with trichomes and pollen grains were tested using the following histochemical tests: iodine iodide solution for starch and proteins, Ruthenium Red for polysaccharides other than cellulose (Johansen 1940; Jensen 1962), Nile Blue for neutral and acidic lipids (Jensen 1962), Sudan III (Johansen 1940) and Sudan Red for total lipids (Brundrett et al. 1991), ferric trichloride for polyphenols (Johansen 1940), potassium dichromate for tannins (Gabe 1968), Nadi reagent for terpenoids (David and Carde 1964), and concentrated sulfuric acid for sesquiterpenes (Cappelletti et al. 1986). The stained sections were observed and photographed with a Nikon Eclipse 400 light microscope.

### Fluorescence microscopy

Pollen grains and fresh hand-made corolla sections with glandular trichomes were analyzed using a fluorescence microscope equipped with filter sets: Cy5 (EX 590–650; BA 663–738), TRITC (EX 525–565; BA 555–600), and FITC (EX 465–495; BA 515–555). Lipophilic substances and essential oils were detected by induction of fluorescence with the Neutral Red fluorochrome (Conn 1953; Lulai and Morgan 1992), steroids with the antimony trichloride fluorochrome (Mace et al. 1974), flavonoids with the aluminum chloride (Guérin et al. 1971), and magnesium acetate (Charrière-Ladreix 1976) fluorochromes. Autofluorescence of secretion of glandular trichomes was observed. Images were acquired with a digital camera Nikon Fi1 and NIS – Elements Br 2 software.

Standard control procedures were conducted simultaneously for all the histochemical and fluorescence methods used, following the recommendations of the respective authors.

### Scanning electron microscopy

Five samples of all flower parts (calyces, corollas, stamens, pistils with nectaries) were fixed in a 4% glutaraldehyde solution in 0.1 M phosphate buffer (pH 7.0) for 12 h at room temperature. Later, the plant samples were rinsed in the same buffer four times and dehydrated in ethanol series (30, 50, 70, 90, 95%), and subsequently three times in absolute alcohol. After dehydration, the plant material was transferred to acetone, dried at critical point in liquid CO_2_ using Bal-Tec CPD 030, and coated with gold using the Polaron SC 7640 sputter coater. The number of nectarostomata within a 1-cm^2^ area of the nectary epidermis and the length and width of ten stomatal pores were measured using morphology software combined with scanning electron microscopy (SEM). The surface of calyces, corollas, stamens, pistils, pollen grains, and nectaries were investigated and depicted at an accelerating voltage of 30 kV using a TESCAN/VEGA LMU scanning electron microscope.

### Quantity of floral rewards

Every year, nectar was sampled with a micropipette and collected on five separate dates of the study period (Jabłoński 2002). Nectar production was determined during the peak of the *L. vulgaris* flowering period (i.e., in late June or the beginning of July). Prior to nectar sampling, the flowers developing on different inflorescences were randomly selected and marked. Then, the inflorescences were bagged (*n* = 26–30 per year and study site) with tulle isolators (mesh size < 1 mm). Twice a day, in the morning (i.e., 7–9 a.m.) and evening (i.e., 7–9 p.m.), we monitored the flowers and noted the progress of flowering. The amount of nectar produced was determined in the bud stage, in 1-day (=when the lower lip folded and the palate started being presented), 2-day, 3-day, and 4-day flowers; each sample contained nectar from 15 to 20 flowers of different individuals. On the appropriate day of anthesis, flowers were removed from the plants and transported into the laboratory (*ca* within 2 hours). Micropipettes with the collected nectar were reweighed (WPS-36 analytical balance RADWAG, Poland). The sugar concentration in the nectar (% *w*/*w*) was determined in each sample using an Abbe refractometer. The mass of the nectar and secreted sugars (in mg) was calculated.

Pollen production was monitored in the full blooming phase. Closed anthers (*n* = 100) were extracted from the flowers. The anthers were placed in tarred glass containers in four replications. The glass containers with collected anthers were inserted into a dryer (Elcon CL 65) for several days, at a temperature ca. 33 °C. After the anthers had burst, the pollen was rinsed from the anthers with 70% ethanol (4–10 ml). The accuracy of the pollen rinsing was checked under a stereomicroscope with a × 5 power. The mass of pollen produced was calculated per flower (Denisow 2011).

### Data analysis

Data are presented as mean values ± SD (standard deviation). We used ANOVA (analysis of variance) to evaluate differences in the mean values of the analyzed features (number of flowers per inflorescence, nectar amount per flower, nectar sugar concentration, sugars mass per flower, pollen mass per flower) between the populations and within the populations between the years of the study. The Tukey HSD test was incorporated for post hoc comparison of means at *α* = 0.05. The data were analyzed using STATISTICA 6.0 (Statsoft Inc.) software.

## Results

### Flowering

**Fig. 1:**
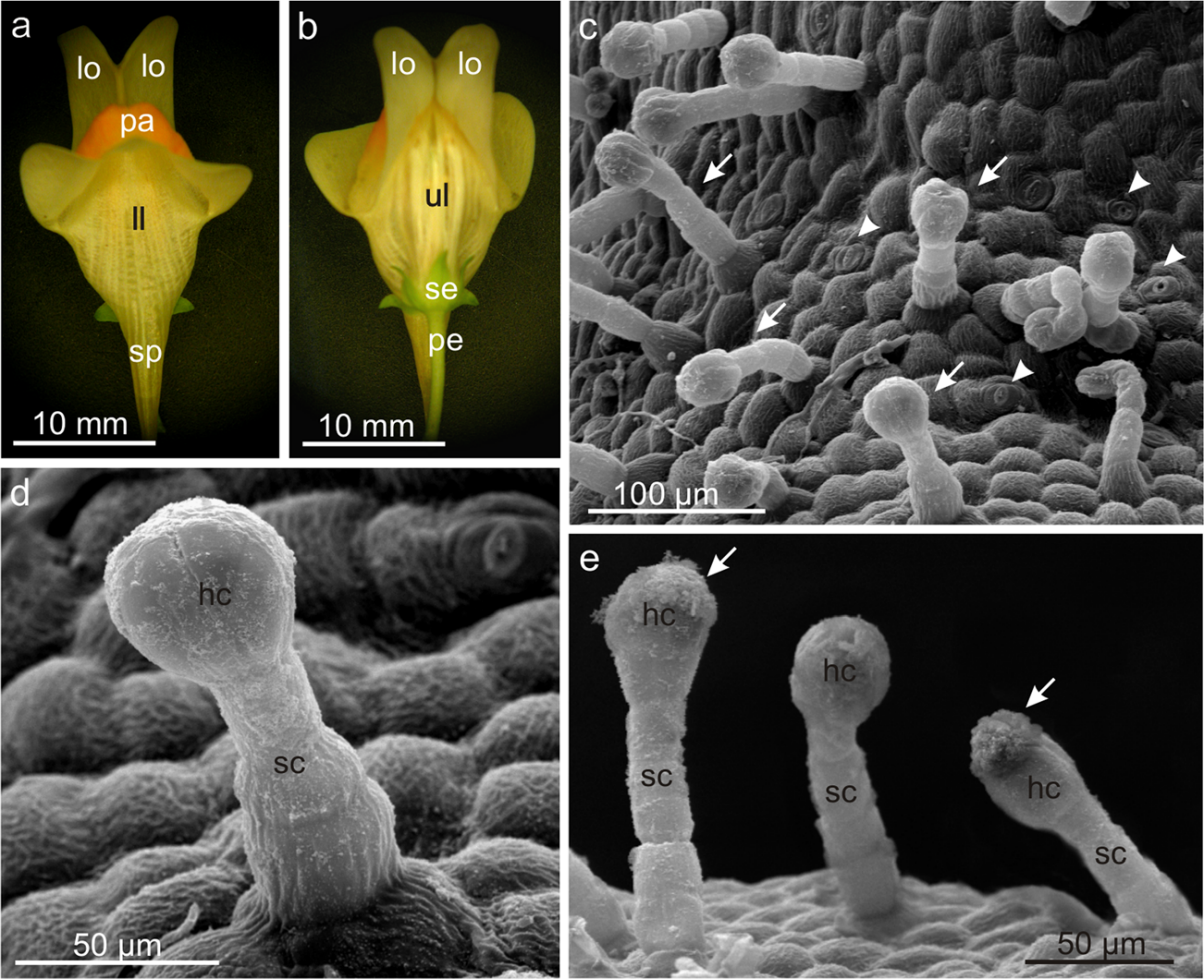
Micromorphology of sepals and peduncle of a *Linaria vulgaris* flower. **a** View of the flower from the side of the lower lip. **b** View of the flower from the side of the upper lip. **c** Surface of the flower peduncle with glandular trichomes (*arrows*) and stomata (*arrowheads*). **d** Glandular trichome on the flower peduncle. **e** Glandular trichome from the sepal with visible secretion (*arrows*). *pe* peduncle, *se* sepals, *ll* lower petal lip, *ul* upper petal lip, *pa* palate, *lo* lobes of the upper lip, *sp* spur, *hc* head cells, *sc* stalk cells

The onset of flowering of *L. vulgaris* was noted during June/July, while the end of blooming was recorded in September/October. Only slight differences (3–4 days) were found in the duration of the blooming period and full bloom phase between the rural and urban populations. However, the full bloom period differed between years of study in the rural population (Table 1). The flowers, arranged in a monopodial inflorescence of the raceme type, developed in acropetal succession (i.e. the lower flowers were older than the upper ones). The zygomorphic flowers of *L. vulgaris* are hermaphroditic (Fig. 1a, b). The number of flowers ranged from 12 to 41 per stem, averaged 34.2 ± 7. In each population, the *L. vulgaris* flower life span was longer in September (3.8 ± 0.6 days) than in July (3.1 ± 0.5 days). The inflorescence life span ranged from 7 to 9 days (mean = 7.8 days).

**Table 1:** The period of blooming of *Linaria vulgaris* in 2013–2014, SE Poland

Population	Year	Blooming period (days)	Full bloom period (days)
Rural (Jastków)	2013	15 June–10 October (118)	67
	2014	23 June–15 October (115)	50
	Mean	116.5	58.5
Urban (Lublin)	2013	19 June–02 October (106)	54
	2014	14 June–10 October (119)	57
	Mean	112.5	55.5

### Flower micromorphology and histochemistry

Numerous glandular trichomes were identified on both the pedicel and the calyx tube fused of 5 sepals (Fig. 1c–e). The glandular trichomes with a multicellular basal foot cells rooted in the epidermis and a 4-celled capitate bulbous (spherical) head were approximately 100 μm long (range of 80–124 μm). Glandular *trichomes produced exudates* composed of lipids, phenolic compounds, and tannins (Table 2).

**Table 2:** Metabolites identified in pollen grains and trichomes of *Linaria vulgaris* flowers by histochemical and fluorescence tests

Staining	Target compounds	Sepal glandular trichomes	Upper lip lobe glandular trichomes	Upper lip lobe papillae	Lower lip non-glandular trichomes	Lower lip papillae	Pollen grains
Sudan III	Total lipids	+	+	+	+	+	+
Sudan Red	Total lipids	+	+	+	+	+	+
Nile Blue	Acid and neutral lipids	+	+	+	+	+	+
Nadi reagent	Terpenoids (essential oils)	–	+	–	–	–	+
Concentrated sulfuric acid	Sesquiterpenes	–	+	–	–	–	–
Ruthenium Red	Polysaccharides other than cellulose	–	+	–	–	+	+
Ferric trichloride	Polyphenols	+	+	–	–	+	–
Potassium dichromate	Tannins	+	+	–	–	–	–
Iodine iodide solution	Starch	–	–	–	–	–	–
	Proteins	–	–	–	–	–	+
Neutral Red under UV	Lipids and essentials oils	nd	+	–	–	+	+
Aluminum chloride under UV	Flavonoids	nd	+	–	+	–	+
Magnesium acetate under UV	Flavonoids	nd	+	–	+	–	+
Antimony trichloride under UV	Terpens contain steroids	nd	+	–	–	–	+

– negative, + positive, *nd* not done

**Fig. 2:**
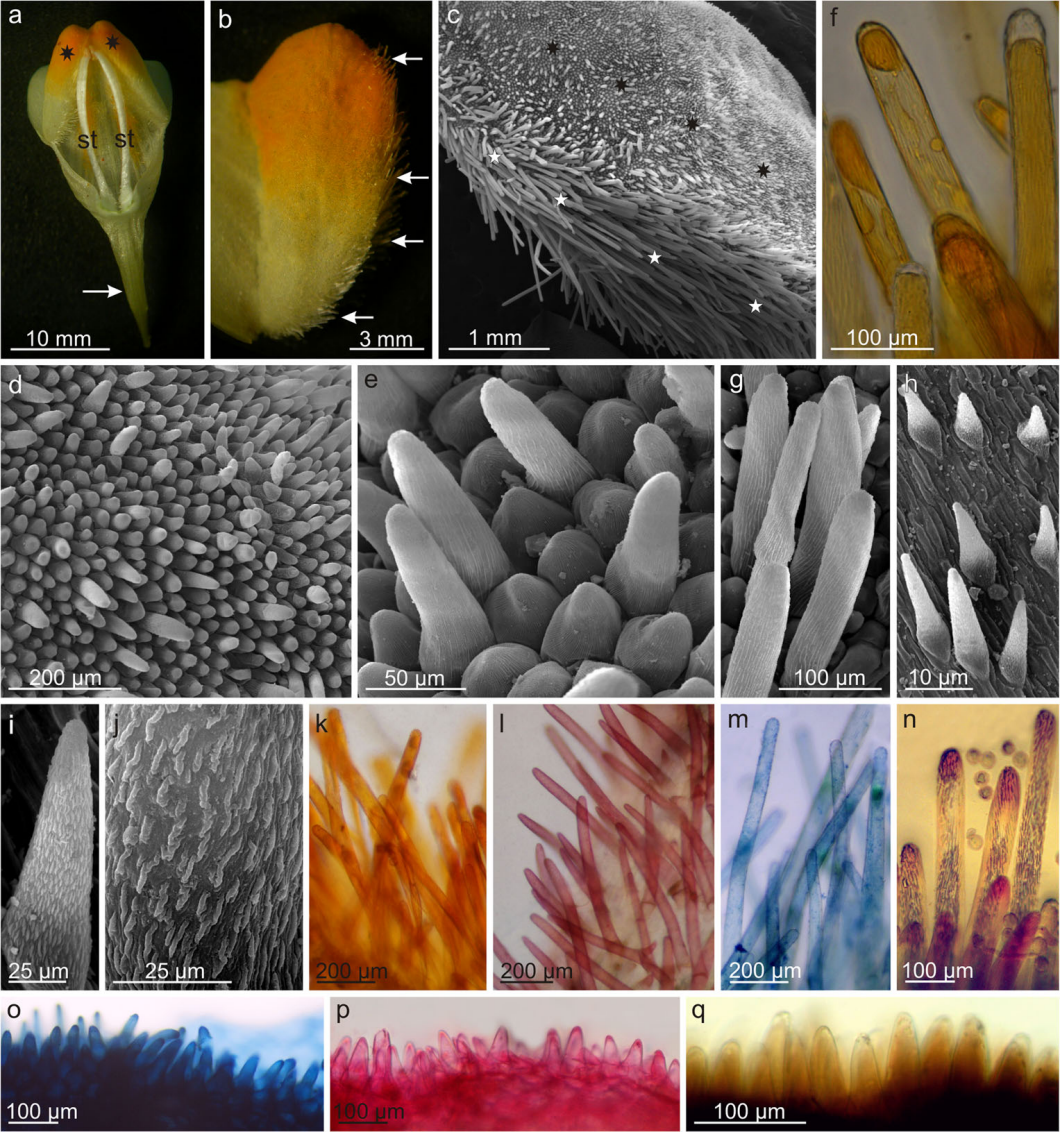
Micromorphology and histochemistry of papillae and non-glandular trichomes from the lower petal lip palate. **a** Longitudinal section through a flower with a visible spur (*arrow*) and palate (*stars*) with orange color. **b** Fragment of the palate with non-glandular trichomes (*arrows*). **c** Surface of the palate with papillae (*black stars*) and non-glandular trichomes (*white stars*). **d**, **e** Papillae (*arrows*) on the palate surface. **f** Non-glandular trichomes with intensive orange pigments in cell vacuoles. **g** Non-glandular trichomes with a striated cuticle. **h**, **i** Conical papillae on the inner surface of the corolla throat. **j** Striations of the cuticle visible on the papilla surface. **k** Non-glandular trichomes after Sudan III staining. **l** After Sudan Red staining. **m** After Nile Blue staining. **n** After ferric chloride staining. **o** Papillae after Nile Blue staining. **p** After Ruthenium Red staining. **q** After potassium dichromate staining. *pa* palate, *sp* spur, *st* stamens

The flowers of *L. vulgaris* emitted a pleasant “honey” scent. The tubular, 2-lipped corolla was brightly yellow due to the presence of flavonoid pigments in its epidermal cells (Fig. 2a, b). The lower lip of the corolla consisted of a 2-lobed, well-developed, orange-colored projection (palate), which obstructed the opening to the throat of the corolla (Figs. 1a and 2a, b). Numerous papillae (50–98 μm long) covered by a striated cuticle were found on the upper surface of the palate (Fig. 2c–e). The papilla cells contained lipids, pectins, tannins, and phenolic compounds (Fig. 2o–q, Table 2). The lower part of the palate formed a shield protecting the stamens. Numerous unicellular or bicellular non-glandular trichomes containing living cytoplasm and yellow pigments in vacuoles were identified on this palate surface (Fig. 2b–g). These trichomes, with a length of approx. 200–609 μm, were covered by regular striated cuticle. Lipids, which exhibited a positive reaction with Sudan III, Sudan Red, and Nile blue, phenolic compounds, and flavonoids were detected in the cells of the non-glandular trichomes (Fig. 2k–n, Table 2).

During the course of anthesis, a change in the palate color was observed. In the bud stage and in 1-day flowers, the palate was yellowish; it was light orange in 2-day flowers and, in 3-day and 4-day flowers, the palate changed color to intensive orange.

At the base of the palate, at the site where the lower and upper petal lips were fused forming the apical part of the corolla throat, conical non-glandular trichomes with an approximate length of 130 μm were observed (Fig. 2h, i). These non-glandular trichomes were directed downwardly into the corolla throat and towards the spur. These trichomes were covered by a cuticle, whose striae formed a specific “stitch” (Fig. 2i, j).

**Fig. 3:**
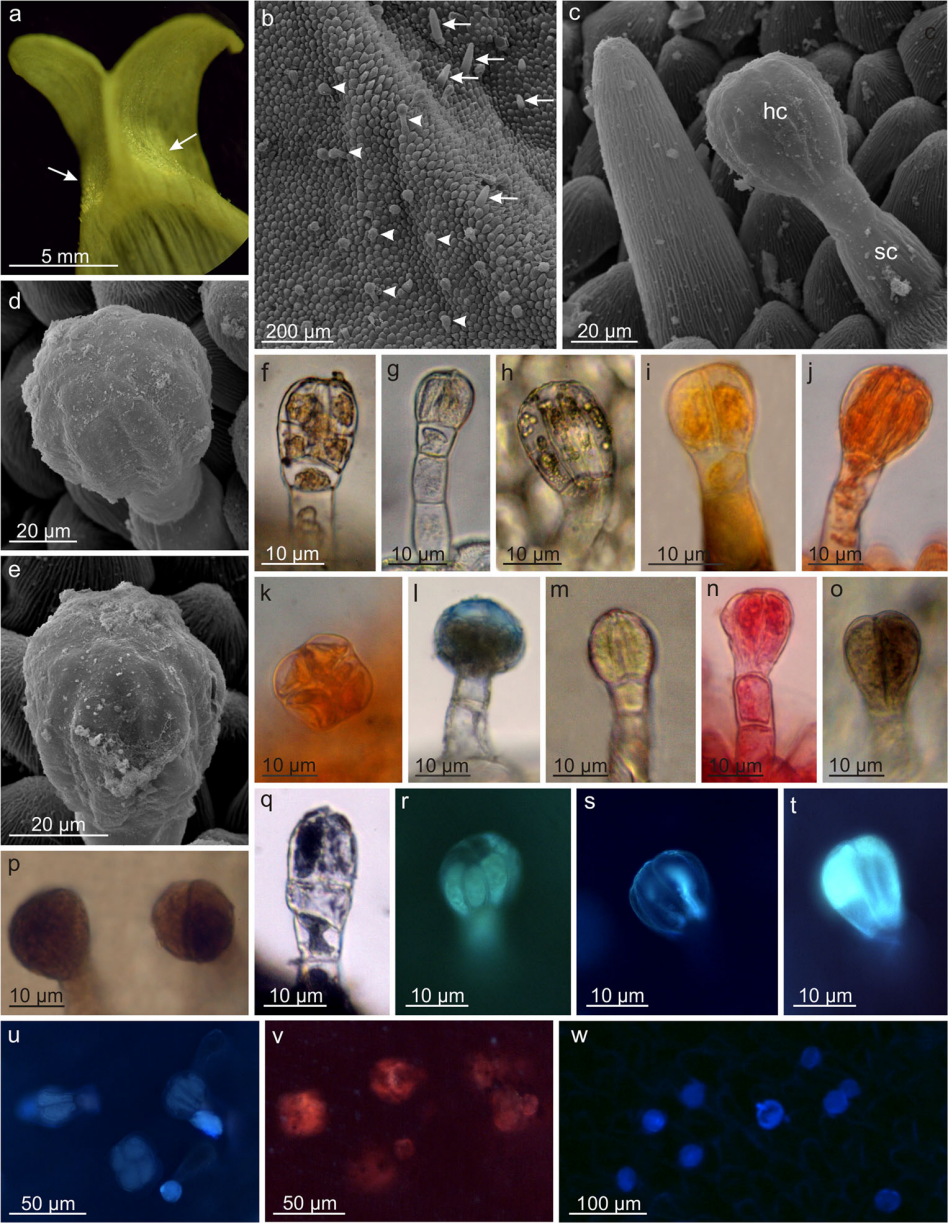
Micromorphology and histochemistry of glandular trichomes on the upper petal lip lobes. **a** Fragment of the upper lip lobe with glandular trichomes (*arrows*). **b** Surface of the upper lip lobe with glandular trichomes (*arrowheads*) and papillae (*arrows*). **c** Glandular trichome and papilla on the lobe surface. **d**, **e** Multicellular heads of glandular trichomes with secretion visible on their surface. **f–h** Different type of glandular trichomes. **f** Trichome with a multicellular several-layer head. **g** Trichome with a four-celled one-layer head. **h** Trichome with a multicellular one-layer head. **i–w** Histochemistry of glandular trichomes. **i–l** Lipids in trichome cells after Sudan III (**i**), Sudan Red (**j**, **k**), and Nile Blue (**l**) staining. **k** Four-celled trichome head, top view. **m** Sesquiterpenes after concentrated sulfuric acid staining. **n** Polysaccharides other than cellulose after Ruthenium Red staining. **o** Tannins after potassium dichromate staining. **p** Polyphenols after ferric chloride staining. **q** Terpenoids after Nadi reagent staining. **r–t** Fluorescence of flavonoids under UV light with magnesium acetate (**r**) and with aluminum chloride (**s**, **t**). **u** Fluorescence of steroids under UV with antimony trichloride. **v** Fluorescence of lipids and essential oils under UV with Neutral Red. **w** Secretion autofluorescence under UV. *hc* head cells, *sc* stalk cells

Additionally, two upwardly folded lobes of the upper lip near the throat opening exhibited many capitate glandular trichomes with a height of approx. 85 μm, composed of a 1- or 2-celled base and a 4- or 8-celled, 1-, 2-, or 3-layered secretory head, as well as a few papillae with a length similar to that of the glandular trichomes located on the palate surface (Fig. 3a–e). Histochemical tests revealed the content of lipids, pectins, phenolic compounds, terpenoids, sesquiterpenes, steroids, and flavonoids, whereas the papillae reacted positively with Sudan III, Sudan Red, and Nile Blue (Fig. 3f–w, Table 2). Furthermore, the glandular trichomes, mainly the secretory head, showed blue autofluorescence under ultraviolet light (Fig. 3w), whereas the papillae showed weak green-blue autofluorescence (not shown).

**Fig. 4:**
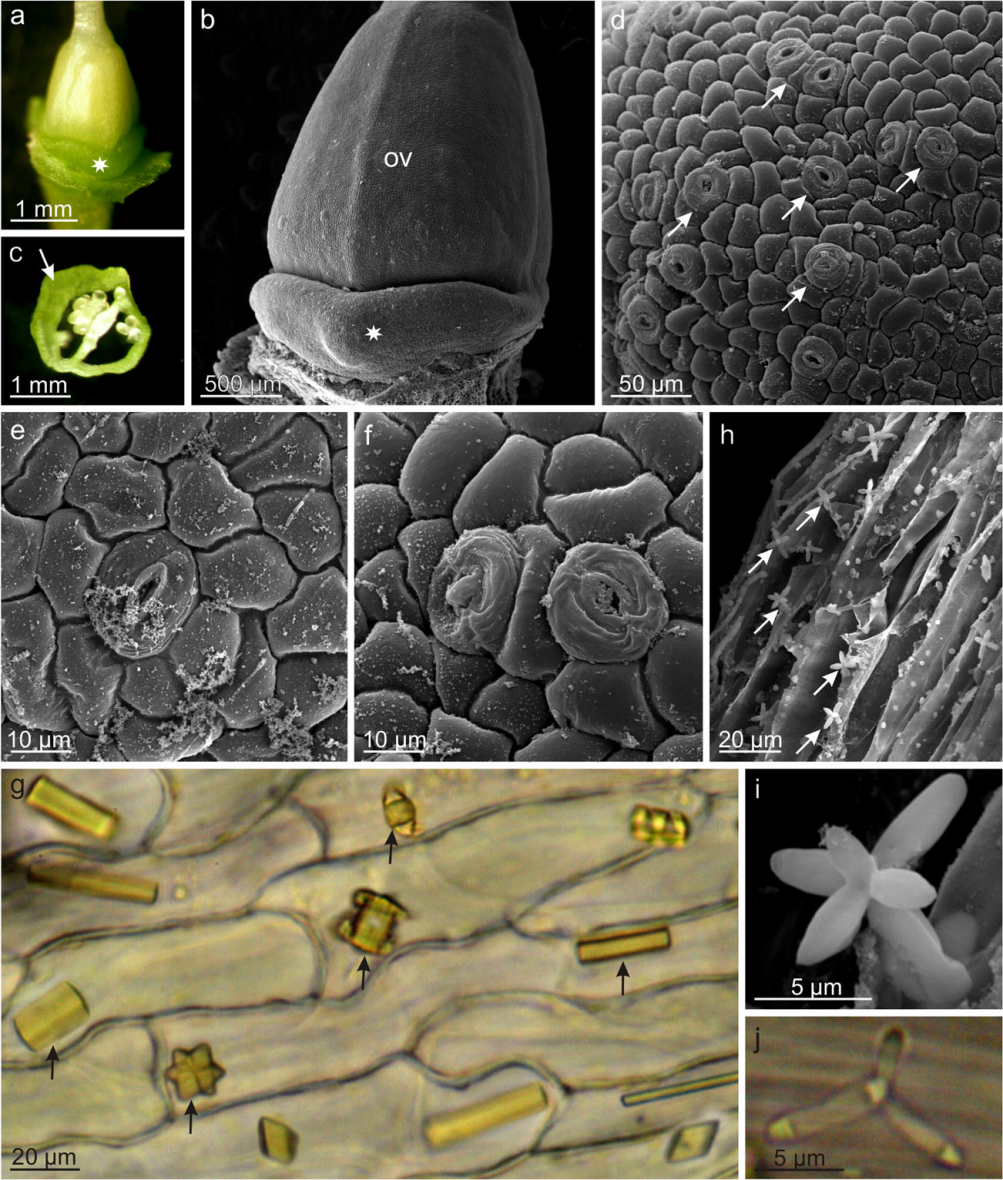
Micromorphology of the nectary and spur in *L. vulgaris* flowers. **a**, **b** Nectary (*stars*) at the base of the ovary visible from the side of the lower lip. **c** Cross-section of the ovary with the nectary (*arrow*). **d** Fragment of the nectary surface with nectarostomata (*arrows*). **e**, **f** Nectarostomata in nectary epidermis with visible secretion. **g** Crystals of calcium oxalate (*arrows*) in the inner epidermis of the spur. **h–j** Yeast cells (*arrows*) visible on the inner epidermis of the spur surface; **i**—side view. **j**—top view. *ov* ovary

The green, fleshy nectary gland in the *L. vulgaris* flowers was located at the ovary base and had a shape of an asymmetrical disc with a diameter of approx. 1.6 mm (Fig. 4a–c). The nectary was the highest at the lower lip (average 444 ± 38 μm) and the lowest on the other side (average 25 ± 7 μm). Nectar is secreted through numerous modified nectarostomata located mainly at the level of the glandular epidermal cells at the side of the lower lip (Fig. 4d–f). On average, there were 475 nectarostomata per mm^2^ of the nectary epidermis; they were characterized by a mean length of 23.5 μm and a width of 17.8 μm.

In the cells of the inner epidermis of the spur, where abundant nectar was accumulated, there were numerous calcium oxalate crystals with various shapes, most commonly appearing as blocks and multifaceted druse crystals (Fig. 4g). In turn, there were many budding yeast cells in the form of 4–5-armed “windmills” observed on the surface of the inner epidermis of the spur (Fig. 4h–j).

**Fig. 5:**
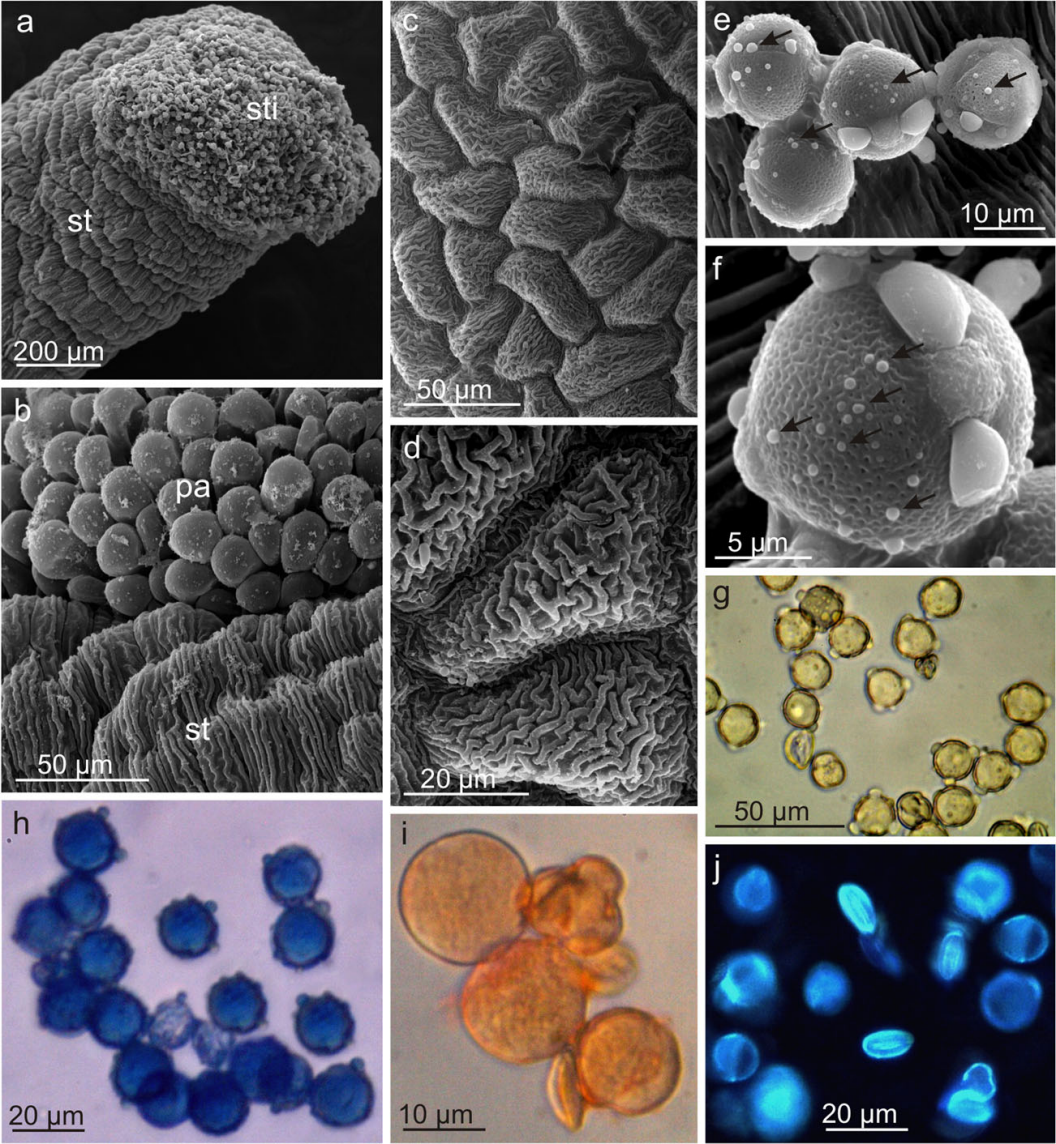
Micromorphology of the generative organs of *L. vulgaris* flowers. **a** Fragment of the style and stigma with visible pollen grains. **b** Striated cuticle on the style surface and papillae on the stigma with flocculent secretion. **c**, **d** Cells of anther epidermis with massive cuticular striations. **e**, **f** Tricolpate pollen grains in SEM; pollenkitt granules visible in the mesh of the reticulate exine (*arrows*). **g** Proteins in pollen grains (reaction with the iodine iodide solution). **h** Terpenoids (Nadi reagent staining). **i** Lipids (Sudan III staining). **j** Flavonoids (magnesium acetate under UV). *st* style, *sti* stigma, *pa* papillae

The stigmata of the 2-carpelled pistil were formed of numerous papillae with a fluffy secretion and germinating pollen grains on their surface (Fig. 5a, b). The style epidermis cells exhibited massive cuticular striae. The pistil was surrounded by 2 longer and 2 shorter stamens, whose filaments were covered by numerous non-glandular trichomes at the base.

The anther epidermal cells had a polygonal shape and, likewise the style, were covered by a striated cuticle (Fig. 5c, d). The pollen grains of *L. vulgaris* were tricolpate with faveolate (reticulate) ornamentation of the exine (Fig. 5e, f). Numerous lipid-protein granules were deposited inside the mesh of the reticulate exine. Additionally, pectins, essential oils, flavonoids, and steroids were evidenced in the pollen grains (Fig. 5g–j, Table 2).

### Nectar and pollen rewards

**Fig. 6:**
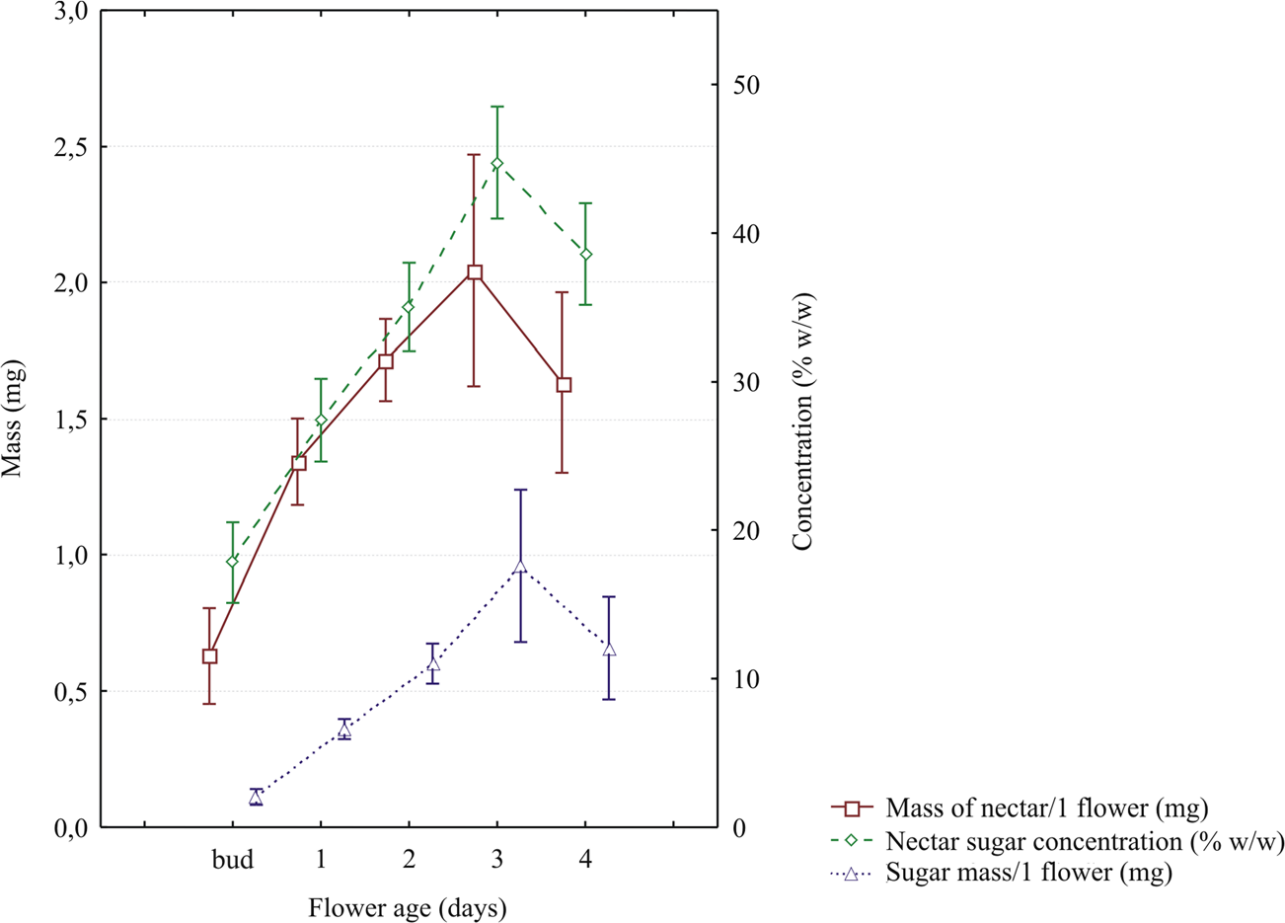
Effect of flower age on nectar mass, nectar sugar concentration and nectar sugar mass in *Linaria vulgaris.* Values are means calculated across the years of study and populations. Vertical bars show 95% confidence intervals

The floral nectar was accumulated in the spur. Nectar release began in the bud stage (approx. 4–6 h before lower lip folding) and lasted to the end of anthesis (i.e., when the flower began to wilt). Significant effect of flower age was found for the mass of nectar per flower (*F*_4,76_ = 22.525, *P* < 0.001), the concentration of sugars in nectar (*F*_4,76_ = 52.714, *P* < 0.001), and for the mass of sugars per flower (*F*_4,76_ = 24.488, *P* < 0.001). Nectar production and nectar sugars concentration increased gradually throughout flower development, peaked in 3-day flowers, and decreased slightly towards the end of flower life span (Fig. 6).

On average, the flowers of *L. vulgaris* from the rural population (*R*) secreted 1.5-fold more nectar (*F*_1,15_ = 8.589, *P* = 0.011) than the flowers of *L. vulgaris* growing in the urban population (*U*) (Table 3). The nectar amount demonstrated a significant year effect (*F*_1,15_ = 9.640, *P* = 0.008). The nectar concentration was high and ranged between 36.5 and 57.0%. Significant year and population effects on the nectar sugar concentration were found (for year *F*_1,15_ = 5.285, *P* = 0.037; for population *F*_1,15_ = 12.846, *P* = 0.003).

**Table 3:** Nectar amount, sugar concentration, and sugar mass per flower at the peak of nectar production (=3-day flowers) in *Linaria vulgaris* in 2013–2014, SE Poland

Population	Year	Nectar amount/1 flower (mg)		Sugar concentration (%)		Sugar mass /1 flower (mg)	
		Mean	SD	Mean	SD	Mean	SD
Rural (Jastków)	2013	2.08_a_	0.32	46.4_a_	4.48	0.96_a_	0.14
	2014	3.54_b_	0.27	54.1_b_	1.98	1.91_b_	0.16
	Mean	2.81_B_		50.3_B_		1.44_B_	
Urban (Lublin)	2013	1.54_a_	0.20	37.9_a_	2.53	0.58_a_	0.03
	2014	2.11_b_	0.36	44.1_a_	3.65	0.94_b_	0.20
	Mean	1.82_A_		41.0_A_		0.76_A_	

Means followed by the same small letters are not significantly different between years and values followed by the same capital letters are not significantly different between study populations at *α* = 0.05 according to the Tukey HSD test

*SD* standard deviation

On average, the total mass of sugar in the nectar was 1.1 mg/flower. The total mass of sugar in the nectar available per flower differed significantly between the populations (*F*_1,15_ = 10.531, *P* = 0.006) and the years of the study (*F*_1,15_ = 9.393, *P* = 0.008).

Dehiscence of *L. vulgaris* anthers began as soon as the palate started being presented. In approximately 10% of the flowers, pollen release was observed in the closed bud stage. No significant population effect was found for the pollen production per flower (*F*_1,15_ = 0.067, *P* = 0.799); however, year-to-year disparities were found (*F*_1,15_ = 16.808, *P* = 0.001). In 2014, the flowers of *L. vulgaris* produced 1.5- to 2-fold more pollen than in 2013 (Table 4).

**Table 4:** Pollen production in flowers of *Linaria vulgaris* in 2013–2014, SE Poland

Population	Year	Mass of pollen/1 flower (mg)		
		Min-max	Mean	SD
Rural (Jastków)	2013	0.14–0.37	0.23_a_	0.09
	2014	0.33–0.50	0.41_b_	0.07
	Mean		0.32_A_	
Urban (Lublin)	2013	0.16–0.29	0.23_a_	0.05
	2014	0.26–0.45	0.37_b_	0.07
	Mean		0.30_A_	

Means followed by the same small letters are not significantly different between years and values followed by the same capital letters are not significantly different between study populations at *α* = 0.05 according to the Tukey HSD test

*SD* standard deviation

**Fig. 7:**
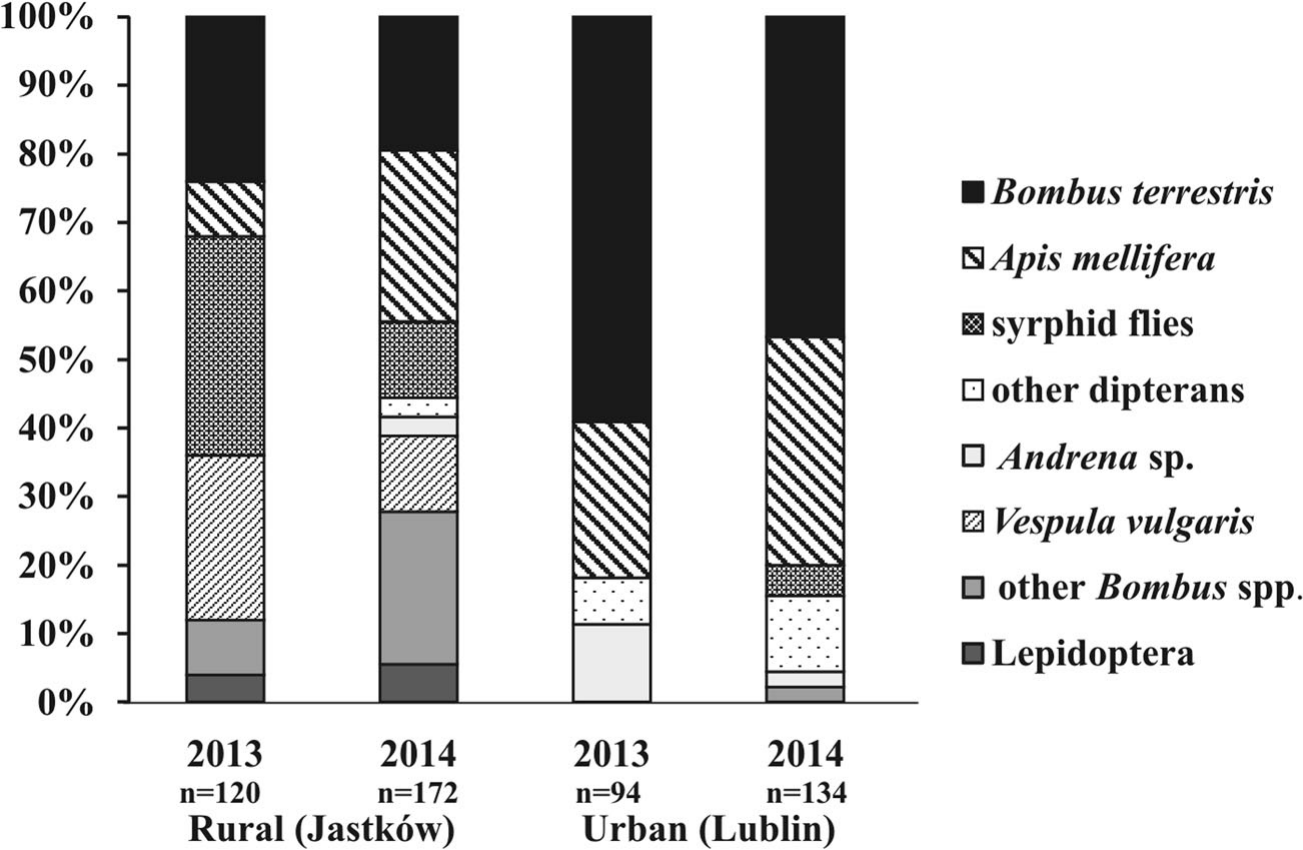
Insect visitors spectrum in *Linaria vulgaris* in two populations in rural and urban landscape in 2013–2014, SE Poland

In good weather conditions (sunny, no precipitation), the flowers of *L. vulgaris* attracted numerous insect visitors, i.e., representatives of Hymenoptera, Diptera, and Lepidoptera (Fig. 7). The insect foraged throughout the day from ca. 6.00 to 19.00 h. The visits of *Bombus* species (including *B. terrestris*, *B. hortorum*, *B. pascuorum*, *B. lapidarius*, *B. sylvarum*) and *Apis mellifera* were distributed quite evenly throughout the day, while dipterans and hymenopterans foraged on the flowers mainly in evening hours. The spectrum of insect visitors differed between the populations and the years of the study. Both richness and abundance of insect visitors were higher in the rural site (Jastków) than in the urban site (Lublin). In the rural population, syrphid flies (33.3% of the visits) and *Vespula vulgaris* (25.0% of the visits each) were most frequently noted in 2013. In the urban population, in both study years, *B. terrestris* (52.9%, on average) and *A. mellifera* (28.0%, on average) were the main visitors.

## Discussion

Insects rely on diverse floral signals (e.g., visual, olfactory) to find flowers and make use of nectar and/or pollen floral rewards (Lunau 2000). We observed insects foraging for both nectar and pollen, which did not support the classical view that *Linaria vulgaris* develops “nectar flowers” (Knuth et al. 1904). We observed insect visitors (mainly bumblebees) foraging for pollen, which is in line with Newman and Thomson (2005b). According to Vargas et al. (2010), *L. vulgaris* is considered as a highly specialized species with nectar reward available only to heavy and long-tongued bumblebees. In our study, both short-tongued (*Apis mellifera*, *Bombus terrestris*, *B. lapidarius*) and long-tongued (*B. hortorum*, *B. pascuorum*, *B. sylvarum*) bees were noted searching for floral nectar in a legitimate way. Moreover, dipterans and solitary bees (e.g., *Andrena* spp.) were recorded; however, these insects were only occasionally able to get inside the flower. This is in agreement with the study of Stout et al. (2000), who often observed small dipterans and syrphids approaching *L. vulgaris* flowers but were not able to collect the nectar. In our study, nectar robbing was observed. *Vespula vulgaris* and *B. terrestris* (about 40% of total individuals) were seen biting holes in spurs (or re-using already bitten holes), which is in line with the observations made by Corbet et al. (1981). *L. vulgaris* develop nototribic flowers and only insects pushing their heads through the corolla entrance in search for nectar can deposit pollen on the stigma. Nepi et al. (2003) found that only long-tongued *B. pascorum* and two Lepidoptera representatives were able to pollinate the flowers in legitimate way, while other insect visitors acted as primary or secondary nectar robbers. Flowers of *L. vulgaris* appear to be “phenotypically specialized” and could be expected to have functionally specialized pollination system, but the spectrum of insect visitors observed in our study suggests to classify the species as “ecological generalist” (Ollerton et al. 2007). We evidenced that the flowers of *L. vulgaris* attract a wide range of insects reaching (or at least trying to reach) nectar from the side of corolla lobes and presumably touching the reproductive organs*.* However, the pollinator effectiveness was not measured, which is the *limitation* of our *research*.

The composition of insect visitors to *L. vulgaris* flowers differed between the urban and rural habitats. This observation indicates that, even in the case of a species with highly specialized flowers (zygomorphic, spurred corolla), the spectrum of floral visitors considerably depends on spatial and temporal variation in the composition and abundance of local insect guild, which is consistent with previous reports (e.g., Kameyama and Kudo 2009; Zych et al. 2013; Antoń and Denisow 2014; Denisow et al. 2014; Ziemiański and Zych 2016).

Moreover, the difference in the composition of insect visitors between the study populations may be explained by the presence or absence of alternative flowering plants in the study site during the flowering of *L. vulgaris*. In the rural site, *Medicago sativa* and *Rorippa sylvestris* were heavily used by bumblebees and honeybees. On the contrary, there was no other attractive co-flowering species in the urban site, which forced bees to visit *L. vulgaris*, i.e., the only flowering species available.

In *L. vulgaris*, the bright orange-colored palate forms a landing platform for pollinators. No doubt, in a flower with a closed corolla and a deep nectar spur, the bright colored landing palate provides pollinators with the information about the route to the nectar. In fact, we observed insects using the palate as a landing platform, crawling inside to reach the nectar, and touching reproductive organs, possibly transferring pollen. Leonard et al. (2013) found that the nectar guides exert an impact on insect behavior, e.g., they can reduce nectar robbing, increase the relative frequency of legitimate visits, and allow insects to save energy while searching for sugar-rich nectar. Therefore, the benefits of nectar guides are potentially shared by both the plant and the pollinator (e.g., Leonard et al. 2013). Moreover, floral pigments that produce patterns attractive to pollinators may also deter florivores (Gronquist et al. 2001). In *Linaria* species, the significant inter-species variation in the color of both the corolla and the palate is suggested to have a high taxonomic significance (Fernández-Mazuecos et al. 2013).

The upper surface of the palate is roofed by non-glandular trichomes and numerous papillae covered with a striated cuticle. These anatomical features of the upper surface of the palate increase friction and provide a perfect surface for insects to hold firmly on the flower. Moreover, the non-glandular trichomes of considerable length present on the palate surface are potentially involved in protection against airborne fungal propagules or dust particles (Mayekiso et al. 2008). The papillae and non-glandular trichomes of a palate store tannins and phenolic compounds, which are likely responsible for flower protection against herbivores and pathogens. Plant pathogens are often transferred between plant individuals by herbivorous insects. The insect repellent activity together with strong antifungal and antibacterial activities of tannins and polyphenols confirmed in several studies (Lattanzio et al. 2006; Montenegro et al. 2013) are considered important for plant protection. Phenolic compounds are also supposed to be involved in UV filtration and can ameliorate the effect of intense summer solar radiation (Morey et al. 2016).

The scent of flowers is considered to be a complex olfactory signal whose function is both to attract pollinators and/or to repeal unwelcome insect visitors and deter herbivores to prevent consumption of reproductive plant structures (Schiestl and Ayasse 2002; Raguso 2008). The flowers of *L. vulgaris* produced an expressive “honey” fragrance. Presumably, the scent was emitted by numerous glandular trichomes located on the upper corolla lip and volatile terpenoids found in the pollenkitt of pollen grains (discussed later). We have demonstrated that the exudates of glandular trichomes contain flavonoids, terpenoids, steroids, sesquiterpenes, polyphenols, and tannins. In particular, terpenoids and sesquiterpenes found in essential oils have long been recognized as a source of plant derived flavors and fragrances (Byers et al. 2014; Hambäck 2016; Lucas-Barbosa et al. 2016). For example, Sutton (1988) and Tekaya-Karoui et al. (2010) recognized a specific pleasant smell similar to violets and/or strawberries in certain species of *Linaria*, and identified 49 volatile components in essential oils of *L. heterophylla* flowers. Terpene-producing trichomes similar to those observed in *Linaria* were also found in the leaves of *Calceolaria adscendens*, a species that is traditionally included in the Scrophulariaceae s.l. (Sacchetti et al. 1999). As reported for the representatives of the genera *Antirrhinum*, *Buddleja*, and *Bartsia* (Scrophulariaceae s.l.), the intensity of floral scent emissions was associated with a subtle change in the corolla color and was able to influence the activity of insect foragers (Pyper 1998; Odell et al. 1999; Wright et al. 2005; Gong et al. 2015). Therefore, it is argued that the floral scent composition could potentially mediate plant-pollinator interactions and can even be useful in prediction of the pollination system. Several other biological activities of essential oils are related to phytoalexins, insect antifeedants, pheromones, defensive agents, allelochemicals, or signaling molecules (Pichersky and Gershenzon 2002). In *L. vulgaris*, the gynoecial ring-like nectary gland with different heights is located at the base of the superior ovary and represents the *nectaria-persistentia* type, characteristic for the representatives of Scrophulariaceae s.l. (Smets 1986; Bernardello 2007). Nectar is released via permanently opened nectarostomata. This species-specific nectar secretion was previously observed by Gaffal et al. (1998) and Nepi et al. (2003).

On average, 1.5-fold more nectar was produced in the flowers of *L. vulgaris* in the rural habitat. The variability in the nectar amount is quite common and can be attributable to diverse environmental factors, i.e., temperature, relative humidity, soil moisture, and soil nutrients (e.g., Petanidou and Smets 1996; Denisow et al. 2014). The amount of nectar production in *L. vulgaris* was reported to be linked to the plant and flower age and differ significantly between geographical localizations (Nepi et al. 2003), which is consistent with our observations. The diversity in the nectar amount can have an impact on pollinators’ behavior and their efficiency in the process of pollen transfer and donation (e.g., Antoń and Denisow 2014).

Regardless of the year of the study and the population, the nectar of *L. vulgaris* was highly concentrated. Such nectar offers great energetic reward and, although it can be relatively difficult to collect and transport, it is considered to be preferred by bumblebees, which can maximize their energy intake and can effectively pollinate the flower (Harder 1986; Nicolson et al. 2013). The pollination of *L. vulgaris* mainly by bumblebees was reported by Stout et al. (2000) and Newman and Thomson (2005b). In our field studies, bumblebees were important visitors of the *L. vulgaris* flowers, in particular in the urban habitat, accounting for ca. 50% of the total insect visitors.

In addition to nectar, *L. vulgaris* offers pollen reward. The amount of produced pollen was similar in both populations (mean = 0.31 mg per flower). However, the pollen productivity of the flowers differed considerably between the years of the study. In 2013, the amount of produced pollen was considerably lower in both populations than in 2014. Microsporogenesis and pollen production are highly attributable to weather conditions, and even empty anthers can develop in unfavorable conditions, e.g., in precipitation deficit (Denisow 2011; Khanduri 2011). In the study region, a significant shortage of rainfalls was noted in 2013, which can be the cause of the decrease in pollen production in *L. vulgaris*.

During the anther dehiscence, the mature pollen grains of *L. vulgaris* were already covered by pollenkitt that formed numerous lipid globules spread over the surface of the exine. This is consistent with the observations reported by Halbritter and Ulrich (2016), who found an electron-dense material of pollenkitt in the interapertural space between the bacula of exine. Pollenkitt that forms a lipidic impermeable layer on the surface of young microspores protects the male gametophytes from dehydration, facilitates their adhesion, protects the protoplast against sunlight, and contributes to adherence of pollen grains to the body of pollinators (Pacini and Hesse 2005). Our histological tests demonstrated that, in addition to lipids and proteins, the pollen grains of *L. vulgaris* contained polysaccharides, flavonoids, terpenoids, and steroids. Several studies have demonstrated that pollen grains of different plant species are a rich source of flavonoids, which are known to be involved in pollen fertility and they are detected exclusively in tapetum cells (Taylor and Grotewold 2005). Pollen derivative flavonoids may also accelerate the pollen tube formation and its germination on the stigma (Wood 2017). Moreover, flavonoids protect the pollen grains from the harmful effects of solar radiation (Flenley 2011) and guard against other abiotic and biotic stress factors (Winkel-Shirley 2002; Pourcel et al. 2007). Volatile compounds (terpenoids) have also been found in pollen grains of *L. vulgaris.* Terpenoids that originate from pollenkitt are involved in emission of odors (Lunau 2000; Pacini and Hesse 2005). In *L. vulgaris*, the combined presentation of visual and olfactory signals derivative from stamens are involved in enhancement of pollen attractiveness to pollinators and positively influence the pollination efficiency (Lunau 2000).

In conclusion, flowers are complex organs that follow the coordination of floral parts, which function together to produce rewards and attract pollinators. A combination of cues (shape, size, color, scent, nectar, and pollen traits) are likely responsible for attraction of insect visitors/effective pollinators and exclusion of ineffective pollinators and/or herbivores in *L. vulgaris.*
